# Hyperbaric oxygen and hyperbaric air treatment result in comparable neuronal death reduction and improved behavioral outcome after transient forebrain ischemia in the gerbil

**DOI:** 10.1007/s00221-012-3283-5

**Published:** 2012-10-02

**Authors:** Michal Malek, Malgorzata Duszczyk, Marcin Zyszkowski, Apolonia Ziembowicz, Elzbieta Salinska

**Affiliations:** 1 Department of Neurochemistry, Mossakowski Medical Research Centre, Polish Academy of Sciences, 5 Pawinskiego Str., 02-106 Warsaw, Poland; 2 Department of Anesthesiology and Intensive Care, Military Institute of Medicine, Warsaw, Poland

**Keywords:** Ischemia, Hyperbaric oxygen therapy, Hyperbaric air, Neuronal damage, Brain temperature, Gerbil

## Abstract

Anoxic brain injury resulting from cardiac arrest is responsible for approximately two-thirds of deaths. Recent evidence suggests that increased oxygen delivered to the brain after cardiac arrest may be an important factor in preventing neuronal damage, resulting in an interest in hyperbaric oxygen (HBO) therapy. Interestingly, increased oxygen supply may be also reached by application of normobaric oxygen (NBO) or hyperbaric air (HBA). However, previous research also showed that the beneficial effect of hyperbaric treatment may not directly result from increased oxygen supply, leading to the conclusion that the mechanism of hyperbaric prevention of brain damage is not well understood. The aim of our study was to compare the effects of HBO, HBA and NBO treatment on gerbil brain condition after transient forebrain ischemia, serving as a model of cardiac arrest. Thereby, we investigated the effects of repetitive HBO, HBA and NBO treatment on hippocampal CA1 neuronal survival, brain temperature and gerbils behavior (the nest building), depending on the time of initiation of the therapy (1, 3 and 6 h after ischemia). HBO and HBA applied 1, 3 and 6 h after ischemia significantly increased neuronal survival and behavioral performance and abolished the ischemia-evoked brain temperature increase. NBO treatment was most effective when applied 1 h after ischemia; later application had a weak or no protective effect. The results show that HBO and HBA applied between 1 and 6 h after ischemia prevent ischemia-evoked neuronal damage, which may be due to the inhibition of brain temperature increase, as a result of the applied rise in ambient pressure, and just not due to the oxygen per se. This perspective is supported by the finding that NBO treatment was less effective than HBO or HBA therapy. The results presented in this paper may pave the way for future experimental studies dealing with pressure and temperature regulation.

## Introduction

Sudden cardiac arrest is a common manifestation of cardiovascular disease affecting a large number of people, and despite advances in resuscitation techniques, the percentage of surviving patients is still very low (Nolan et al. 2008). The mortality in patients who achieve return of spontaneous circulation (ROSC) is due largely to post-cardiac arrest syndrome which comprises anoxic brain injury, post-cardiac arrest myocardial dysfunction, ischemia/reperfusion response and persistent precipitating pathology (Nolan et al. 2008). Among these anoxic brain injury is a major cause of mortality, responsible for approximately two-thirds of deaths. The mechanisms of brain tissue damage triggered by cardiac arrest and resuscitation include energy deficit, anoxic depolarization, excitotoxicity, disturbed calcium homeostasis, formation of free radicals and activation of cell death signaling pathways (Busl and Greer 2010). Currently, hypothermia is the only proven treatment for anoxic brain injury after cardiac arrest, and although some drugs appear to have short-term benefits, no specific drug therapy has been confirmed to improve long-term survival in randomized controlled trials (Morley 2011; Deakin et al. 2010).

There is a lot of recent evidence that hyperbaric oxygen (HBO) prevents neuronal damage and improves neurological outcome after cardiac arrest or stroke (for review see Harch and Neubauer 2009), and there is the opinion that amount of oxygen delivered to the brain in a short time after cardiac arrest or after stroke may be an important factor in preventing neuronal damage resulted from brain ischemia (Neumar 2011; Rosenthal et al. 2003; Van Meter et al. 2008). The neurological outcome after cardiac arrest may be greatly affected by the oxygen inhaled immediately after resuscitation, and there is a growing body of evidence suggesting that delivering too much oxygen too quickly may increase injury associated with postischemic reperfusion. There are reports that postresuscitation hyperoxemia (PaO_2_ ≥ 300 mmHg) exposure in the first 60 min after ROSC increased the mortality in patients and was associated with lower independent functional status among patients who survived (Kilgannon et al. 2011).

Despite many reports on beneficial results of HBO in global cerebral ischemia/anoxia in animal models and human clinical studies (for review see Harch and Neubauer 2009), this therapy did not find the common use in neurological diseases treatment. Pressure-related complications of HBO and growing concern that HBO treatment may generate toxic free radicals, resulted in skepticism about the safety and efficacy of HBO in cerebral ischemia (Kot and Mathieu 2011). Nevertheless, HBO still remains the subject of interest of many scientists: moreover, the critical analysis of negative results and data from new animal studies renewed the interest in HBO therapy (Michalski et al. 2011). Recent publications have provided essential information about the important factors influencing HBO efficacy such as the therapeutic time window and the mechanism of action (Rockswold et al. 2010; Lou et al. 2004). It is now known that HBO not only significantly increases oxygen pressure and concentration in the arteries, resulting in better oxygen supply to the ischemic areas, but also increases cerebral blood flow (CBF) into the injured brain, the so-called inverse steal phenomenon mediated by nitric oxide (NO) (Lassen and Palvölgyi 1968; Atochin et al. 2003; Dean et al. 2003). HBO also ameliorates blood flow velocity due to increased deformability of red blood cells (van Hulst et al. 2003).

There is now increasing experimental evidence that HBO can afford neuroprotection after experimental global cerebral ischemia induced by vascular occlusion. It has been shown that HBO treatment results in the reduction in neuronal death and accelerated neurological recovery in different animal models of global ischemia (Iwatsuki et al. 1994; Kapp et al. 1982; Krakovsky et al. 1998; Takahashi et al. 1992) and also in an experimental cardiac arrest model in dogs (Rosenthal et al. 2003). In addition, HBO improves also the rate of return of spontaneous circulation after prolonged cardiopulmonary arrest (Van Meter et al. 2008), decreases lactate concentration in cerebrospinal fluid, improves mitochondrial function after focal cerebral ischemia (Rockswold et al. 2001; Daugherty et al. 2004), triggers multiple neuroprotective effects such as reduction in concentration of cyclooxygenase-2 (COX-2) (Yin et al. 2002) and intracellular adhesion molecule-1 (ICAM-1) (Buras et al. 2000), and decreases the expression of proapoptotic genes including hypoxia inducible factor-1α (HIF-1 α), p53, caspase-9 and caspase-3 (Li et al. 2005). It was also shown that HBO may cause hypothermia due to increased oxygen partial pressure (Fenton et al. 1993).

Regardless of these beneficial effects of HBO on brain tissue, the consequences of forced oxygen application on other organs have to be considered. Lung toxicity is a known phenomenon following exposure to a high oxygen concentration. Prolonged exposure to hyperoxia resulting from HBO and from normobaric oxygen (NBO) can damage pulmonary epithelial cells, and concomitantly occurring chronic pulmonary diseases are additional contraindications in the use of oxygen in cerebral ischemia (Li et al. 2007; Sinclair et al. 2004).

The application of increased oxygen to ischemic areas dates from the mid-1900s and in the early experiments compressed air was used in hyperbaric medicine (for review see Singhal 2006), and later on Smith et al. (1961) and Illingworth (1962) showed the beneficial effect of HBO on cerebral ischemia. To date there is little data showing the effect of hyperbaric air (HBA) on postischemic survival. HBA is not used even as a control to compare the effects of HBO. The few papers containing data about the effects of HBA treatment on brain tissue concern the hypoxic tolerance induced by hyperbaric preconditioning or the impact on cell viability of hypoxic brain slices (Peng et al. 2008; Günther et al. 2004). Recent publications showing that the beneficial effect of HBO may not be the only result of increased oxygen supply (Rosenthal et al. 2003; Miljkovic-Lolic et al. 2003) suggest that the mechanisms of hyperbaric prevention of brain damage still remain unclear.

The aim of our study was to compare the effects of HBO, HBA and NBO treatment on gerbil brain condition after transient forebrain ischemia as a model of cardiac arrest, by testing neuronal survival in the hippocampus, changes in the brain temperature and also basic behavior in the nest-building test. Additionally, possible differences dependent on the time of initiation of the hyperbaric therapy were investigated.

## Experimental procedures

### Animals

**Fig. 1:**
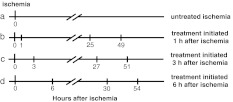
Scheme of the procedure of experiments including untreated 3-min ischemia (**a**) and HBO, HBA or NBO treatment initiated at different times after ischemia (**b**, **c**, **d**). Each time point indicates initiation of the treatment lasting 60 min; treatments were repeated in 24-h intervals (for details of the protocol see section “Experimental procedures”)

All the experiments used male Mongolian gerbils (Meriones unguiculatus), bred in the Animal Colony of the Medical Research Centre, Polish Academy of Sciences in Warsaw, aged 12–13 weeks and weighing about 60 g. The animals were kept at the room temperature (24–25 °C), fed ad libitum and randomly assigned into experimental groups (group 1—sham operated, group 2—non treated ischemia, group 3—ischemia + HBO, group 4—ischemia + HBA and group 5—ischemia + NBO). The animals from groups 2—5 were submitted to a 3-min forebrain ischemia, followed by either hyperbaric therapy or normobaric oxygen application, started at three different times after ischemia (Fig. 1). Sham-operated animals served as controls in evaluation of neuronal cell loss and in behavioral experiments. Animals used in nest-building test experiments served also for neuronal loss evaluation. The number of animals per experimental group ranged from *n* = 5 to *n* = 9 (exact number given in figure legends). The total number of animals used in this study *n* = 120. All animal experiments were carried out according to the Polish and European Community Council regulations concerning experiments on animals.

The animal experiments were approved by the Local Ethical Committee in Warsaw, Poland, and performed in accordance with Polish governmental regulations (Dz.U.97.111.724) and the European Community Council Directive of 24 November 1986 (86/609/EEC). All efforts were made to minimize animal suffering and the number of animals used.

### Forebrain ischemia

The brain ischemia was performed as described earlier (Chandler et al. 1985; Duszczyk et al. 2006b). Briefly, the animals were anesthetized with 4 % halothane in a gas mixture containing 30 % O_2_ and 70 % N_2_O, and 2 min before the operation, the concentration of halothane was reduced to 2 % and kept at this concentration during ischemia. The carotid arteries were isolated through an anterior middle cervical incision, made after the injection of local anesthetics. Cerebral ischemia was induced by the occlusion of both common carotid arteries with miniature aneurismal clips for 3 min. Sham-operated animals were exposed to similar surgery without carotid artery occlusion. During the surgery, animals were kept on the heating pad at 37 °C. After wound closure animals were put into separate cages and moved to the room designed for behavioral test or brain temperature measurements accordingly. Both groups of animals were then submitted to hyperbaric therapy or normobaric oxygen. In this study, ischemic protocol resulted in less than 3 % mortality and there was no mortality that could be associated with hyperbaric or normobaric oxygen treatment.

### Hyperbaric treatment

Animals subjected to ischemia were divided into three groups. Animals from group 3 were treated with HBO therapy (compression in 100 % oxygen), group 4 was compressed in atmospheric air (HBA) and group 5 consisted of animals treated with normobaric oxygen (100 % oxygen at 1 ATA). As a control served sham-operated animals treated with HBO. Animals were placed into the hyperbaric chambers (for HBO—Model B-11 Animal Research Chamber, Reimers Systems, INC., USA; for HBA—Hyperbaric Chamber, Technika Podwodna, Poland) for 60 min 1, 3 or 6 h after ischemia and compressed to 2.5 ATA (rate of compression and decompression 1 ATA/min). Animals from the NBO group were placed in an unpressurized hyperbaric chamber with a constant 100 % oxygen flow through the chamber at a rate of 0.5 l/min. The treatment was repeated for 3 following days. The temperature in the hyperbaric chambers was monitored during the whole session and was maintained at 24–25 °C.

### Measurement of brain temperature

For continuous recording and analysis of the brain temperature in conscious and freely moving gerbils, a telemetric system to measure the brain temperature was utilized (Mini Mitter VitalView hardware and software system, Mini Mitter Co. Inc. Oregon, USA). The temperature was measured in gerbils submitted to 3-min forebrain ischemia and treated with HBO, NBO and HBA. The procedure for the brain temperature probe (probe type Mini Mitter XM-FH-BP) implantation has previously been described in detail (Duszczyk et al. 2006a). Briefly, the holders of the brain temperature probes were implanted unilaterally in gerbils submitted to halothane anesthesia and the tips into the striatum, approximately to the same depth as the hippocampus. Two days later, the probes were inserted into the probe holders and the gerbils were placed in plastic cages resting on the telemetry receiver. Temperature measurements began 3 h before an ischemic episode and continued for 72 h. The temperature signals from the probe were sampled every 30 s, to ensure strict control of the temperature. The mean temperatures were calculated for individual time points (usually every 1 h). Because of technical limitations, brain temperature was not measured during hyperbaric sessions (around 60 min) and the measurements were continued immediately after returning the animals from hyperbaric chamber into the cages. It was shown earlier that insertion of small temperature probe into brain caused only minor injury that did not affect the main results (Duszczyk et al. 2006a; Nurse and Corbett 1994; Zhang et al. 1997).

### Histochemistry

One week (for TUNEL staining) or 2 weeks (for cresyl violet staining) after ischemia, animals were killed for brain examination (3–5 from each experimental group). This time was chosen according to earlier observations that after transient forebrain ischemia, most of the apoptotic processes in gerbil brain is finished, whereas one week after ischemia apoptosis is still observed (Ko et al. 2009; Goda et al. 2012). Animals were anesthetized with halothane and subjected to intracranial perfusion fixation with 4 % neutralized formalin (Sigma-Aldrich, St. Louis, Missouri, USA). The brains were removed and immersed in 4 % formalin for 1 week, then transferred to absolute ethanol and embedded in paraffin. Ten-μm cross sections from the dorsal part of the hippocampus (between 2.2 and 3.5 mm posterior to bregma) were used to evaluate the brain damage size and hippocampal apoptotic neurons. Sections were stained with cresyl violet (Sigma, St. Louis, Missouri, USA) or terminal deoxynucleotidyl transferase-mediated dUTP-nick end labeling (TUNEL, In Situ Cell Death Detection Kit, Fluorescein; Roche, Switzerland). For each animal, at least 5 sections of the central part of CA1 region in both hippocampi were analyzed for neuron density or TUNEL stained cells (number of animals per group *n* = 3–5). The number of neurons was counted in central CA1 area of 0.5 mm length using AxioVison imaging program (Carl Zeiss, Aalen, Germany). A mean number of neurons stained with cresyl violet were expressed as a percentage of mean number of neurons of sham-operated gerbils. The mean number of pyramidal neurons in CA1 region of sham-operated gerbils amounts to 155 per 0.5 mm.

### Nest-building test

Nest-building behavior was evaluated in gerbils submitted to 3-min forebrain ischemia (group 2) and those treated after ischemia with HBO, HBA and NBO (group 3, 4 and 5, respectively) starting at different times after operation (1, 3 and 6 h). Additional groups consisted of sham-operated gerbils (group 1) exposed to HBO, HBA and NBO at the same times as the ischemic groups.

After surgery, each animal was placed into a cage covered with a paper towel and the nest building was assessed each day for 7 days following the ischemic episode. Paper shredding was scored on a 4-point scale adapted from Babcock et al. (1993): 0 = none; 1 = pieces >4 cm^2^; 2 = pieces between 2 and 4 cm^2^; 3 = pieces <2 cm^2^ (Duszczyk et al. 2006a). Some of these animals were subsequently used for evaluation of neuronal damage.

### Statistical analysis

Apart from nest-building scores, the results are expressed as mean ± SEM for each experimental group. Statistical analysis of that data was performed by one way ANOVA, with further analysis using the post hoc least significance test for significant differences between groups (GraphPad Prism, version 5.01; GraphPad Software Inc., La Jolla, California, USA). Differences were considered significant with p value less than 0.05.

The nest-building scores are presented as medians, estimated by calculation of the interquartile range (IQR) and tested by two-way analysis of variance (ANOVA).

## Results

### HBO and HBA prevent neurodegeneration

**Fig. 2:**
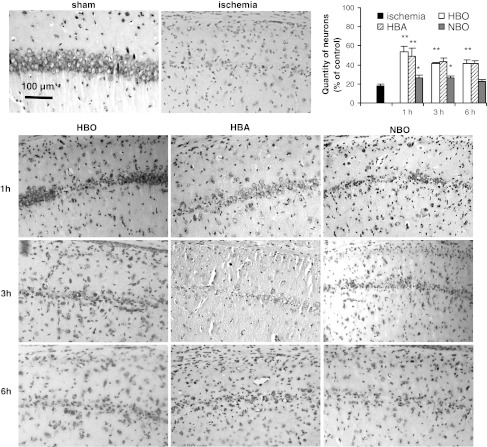
The effect of HBO, HBA and NBO treatment initiated at different times after ischemia (1, 3 and 6 h) on number of neurons in hippocampal CA1 region of the gerbil brain, stained with cresyl violet. The hyperbaric and NBO treatment was applied 3 times in 24-h intervals. Brain tissue was examined 14 days after ischemia. *Top right* corner graph represents results expressed as percentage of surviving neurons compared to the mean control level of 290 cell/mm in central CA1 region in sham-operated animals. Number of analyzed animals per group *n* = 5. Results on the graph are mean values ± SEM; *p < 0.05, **p < 0.001

**Fig. 3:**
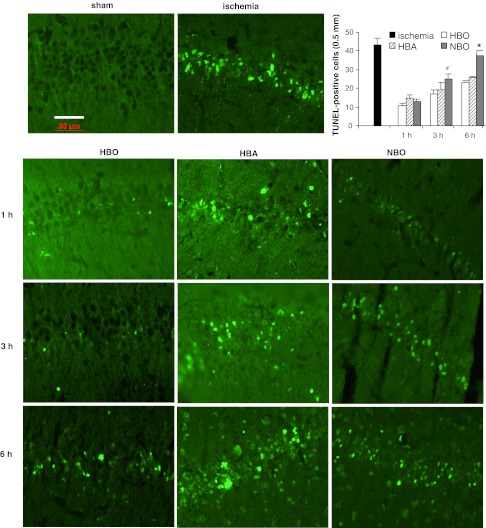
TUNEL-positive cells detected in CA1 region in HBO, HBA and NBO treated animals. Treatment was initiated at different times after ischemia (1, 3 and 6 h). The hyperbaric and NBO treatment was applied 3 times in 24-h intervals. Brain tissue was examined 7 days after ischemia. *Top right* corner graph represents results expressed as number of TUNEL-positive cells in the central CA1 area of 0.5 mm length. Number of analyzed animals per group *n* = 3–4. Results on the graph are mean values ± SEM. *—significantly different from HBO and HBA groups, *p* < 0.001; ^#^—significantly different from HBO group, *p* < 0.01

The counting of pyramidal neurons in CA1 area of hippocampus showed that 3-min ischemia resulted in a significant, 82 % neuronal loss in comparison with control, sham-operated animals (*F*_1,15_ = 135, *p* < 0.001) (Fig. 2). Ischemia-initiated apoptotic processes and one week after ischemic insult 45 TUNEL-positive cells in examined CA1 area (0.5 mm length) were observed (Fig. 3). The application of HBO treatment 1 h after ischemia increased the number of neurons observed in this region to 54 % of control (significant difference from ischemia, *F*_1,26_ = 21.19, *p* < 0.001) (Fig. 2). Application of HBO 3 or 6 h after ischemia resulted in significant increase in number of neurons to 41 % in both cases (*F*_1,14_ = 92.2, *p* < 0.001 and *F*_1,14_ = 60.38, *p* < 0.001, respectively). HBO also significantly reduced the number of TUNEL-positive cells observed in CA1 by 80, 62 and 49 %, respectively, to the time of treatment initiation (*F*_3,13_ = 25.1, *p* < 0.001) (Fig. 3) The use of HBA also significantly increased the number of neurons (Fig. 2). Application of HBA 1 h after ischemia resulted in an increase in viable neurons to 49 % (*F*_1.12_ = 29.24, *p* < 0.001), and HBA therapy applied 3 or 6 h after ischemia in 43 and 41 % of control, respectively (*F*_1,12_ = 45, *p* < 0.001 and *F*_1,15_ = 71.1, *p* < 0.001). In comparison with untreated ischemia, HBA treatment decreased the number of TUNEL-positive cells by 67 % when initiated 1 h after ischemia and by 58 and 40 % when initiated 3 and 6 h after ischemia, respectively (*F*_3,10_ = 13.2, *p* < 0.001). Statistical analysis of results comparing HBO and HBA therapy did not reveal significant differences between their effectiveness. The application of NBO 1 and 3 h after ischemia resulted in only a slight increase in the number of surviving neurons (29 and 26 % in both times, respectively, compared to 18 % of surviving neurons in non-treated ischemia; *F*_1,14_ = 7.63, *p* < 0.05 and *F*_1,27_ = 9.28, *p* < 0.01, respectively), which was significantly lower compared to HBO and HBA (*F*_2,34_ = 5.9, *p* < 0.01 and *F*_2, 25_ = 11.6, *p* < 0.001, respectively). NBO applied 1 h after ischemia reduced the TUNEL-positive cells number by 70 % and by 42 % when initiated 3 h after ischemic insult (*F*_1,8_ = 39.9, *p* < 0.001 and *F*_1,8_ = 11.6, *p* < 0.01, respectively). The application of NBO at 6 h resulted neither in significant increase in number of neurons observed in CA1 region of hippocampus nor in decrease in TUNEL-positive cells (Figs. 2, 3).

### The effect of HBO and HBA on gerbil brain temperature

**Fig. 4:**
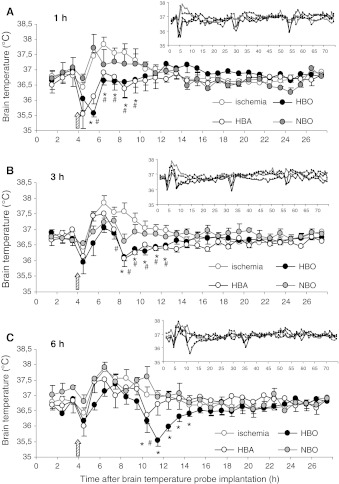
Effect of HBO, HBA and NBO treatment started 1 h (**a**), 3 h (**b**) and 6 h (**c**) after ischemia on gerbil brain temperature. The hyperbaric and NBO treatment was applied 3 times in 24-h intervals. Main graphs show temperature measurement during first 27 h. Brain temperature was measured for 74 h; complex results are presented on small graphs. Three-min ischemia was induced at 4th hour after probe implantation (*arrow*). Results are mean values ± SEM; *n* = 4–5 per each group. *—HBO group significantly different from untreated ischemia, *p* < 0.01; ^#^—HBA group significantly different from untreated ischemia, *p* < 0.01

Brain temperature measurements showed that the mean temperature of gerbil brain after implantation of the probe varied between 36.5 and 37 °C. A significant increase, up to 37.8 °C, in brain temperature was observed up to 4 h after ischemia which remained through the whole measurement period. The application of HBO therapy 1 or 3 h after ischemic incident resulted in a significant decrease in brain temperature (*F*_1,14_ = 5.03, *p* < 0.04 and *F*_1,20_ = 17.9, *p* < 0.001, respectively) and was observed up to 10 h after HBO treatment; subsequently, the temperature remained in the range registered before ischemia (Fig. 4a, b). HBO applied 6 h after ischemia also prevented brain temperature increase (*F*_1,20_ = 22.16, *p* < 0.001) (Fig. 4c). The application of HBA to the ischemic gerbils 1 or 3 h after ischemia also resulted in a significant decrease in the brain temperature (*F*_1,20_ = 19.5, *p* < 0.001 and *F*_1,18_ = 7.56, *p* < 0.05, respectively) and was observed for up to 10 h after the first HBA treatment (Fig. 4a, b); conversely, the application of HBA 6 h after ischemic incident resulted in only a slight, insignificant decrease in brain temperature and was only observed up to 3 h after treatment (Fig. 4c). Hyperbaric sessions themselves had an influence on the temperature of the brain, and after each session an additional, significant transient drop of temperature was observed.

NBO treatment to ischemic gerbils resulted in a significant decrease in brain temperature only when NBO was applied 1 h after ischemia and measured temperatures were significantly different from the ischemic control only up to 3 h after NBO (*p* < 0.01). Application of NBO 3 and 6 h after ischemia produced a short transient decrease which disappeared when NBO treatment was terminated (Fig. 4).

### The effect of HBO and HBA on nest-building behavior in ischemic gerbils

**Fig. 5:**
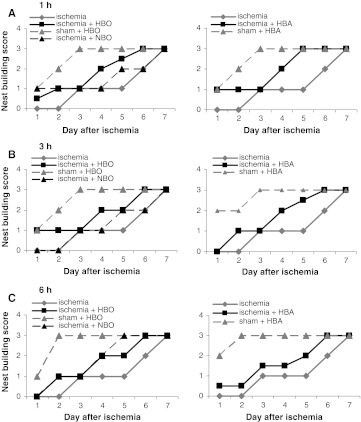
Nest-building behavior in gerbils after HBO, HBA and NBO treatment started at 1 h (**a**), 3 h (**b**) and 6 h (**c**) after ischemia. The hyperbaric and NBO treatment was applied 3 times in 24-h intervals. Nest building was assessed each day for 7 days following ischemic episode. Values are median nest-building score, IQR for each data point ≤1. Group size: sham *n* = 5; ischemia *n* = 9; HBO, HBA and NBO each time group *n* = 5

The nest-building behavior of gerbils was monitored for 7 days following the ischemic insult. Our current and previous experiments showed that naïve and sham-operated animals start nest building immediately after placing them into the experimental cage (Duszczyk et al. 2006a), and after 3-min ischemia, the gerbils exhibited a 2-day delay in the initiation of nest building and a paper shredding score of 3 was reached at the end of the observation period (day 6 or 7) (Fig. 5). In the present experiment, sham-operated animals were treated with HBO and HBA, which did not significantly influence the nest-building behavior. In all experimental groups, the nest-building process improved over time but significant differences in the time required to reach the highest score were observed.

The HBO therapy in gerbils submitted to 3-min ischemia resulted in significant improvement in the nest-building scores. The animals which received the HBO therapy 1 or 3 h after the insult started to build the nest within 24 h after ischemia, and the nest-building process was significantly improved compared to non-treated animals (*F* = 22.28, *p* < 0.001 for HBO and *F* = 22.02, *p* < 0.001) (Fig. 4a, b). The delay of nest building in animals treated with HBO 6 h after ischemia was only 1 day, but the difference in behavior of treated and non-treated animals was significant (*F* = 18.65, *p* < 0.001) (Fig. 5c). Animals treated with HBA also showed significant improvement in nest-building behavior compared to non-treated (*F* = 18.43, *p* < 0.001 for HBA treatment started 1 h after insult, *F* = 21.57, *p* < 0.001 and *F* = 21.32, *p* < 0.001 for treatment initiated 3 and 6 h after ischemia, respectively), and this was not significantly different from that observed after HBO. NBO treatment initiated 1 and 6 h after ischemia also resulted in quicker nest building (*F* = 27.40, *p* < 0.001 and *F* = 33.07, *p* < 0.001, respectively) (Fig. 5a, c), although the NBO treatment applied 3 h after ischemia did not.

## Discussion

The results presented in this paper demonstrate that both HBO and HBA, applied up to 6 h after transient forebrain ischemia in gerbils, induce morphological protection of the CA1 pyramidal neurons and also reduce behavioral deficits in the nest-building task to a higher degree than the application of NBO.

The CA1 pyramidal neurons both in experimental animals and in humans undergo selective cell death after transient forebrain ischemia (Bonnekoh et al. 1990). This phenomenon is termed “delayed neuronal death” because pyramidal neurons of CA1 mostly remain unchanged at the early stage after the event but begin to die in a time window of usually 3–4 days after ischemia due to apoptotic processes (Nitatori et al. 1995). As known from experimental research, the CA1 pyramidal neurons of the gerbil hippocampus degenerate within 2–4 days after 5-min transient global ischemia and it was reported that only 5.8 % of the neurons survive 3 weeks later (Bonnekoh et al. 1990). Our results show that one week after an ischemic insult, lasting 3-min apoptotic processes were still in progress, and after 2 weeks, only 18 % of CA1 pyramidal neurons were intact. HBO therapy significantly reduced the number of dying neurons, and protection was most effective when HBO was initiated 1 h after ischemia; however, the therapeutic window for HBO was still open 6 h after ischemia. These results are in agreement with previously published data reporting a therapeutic window for HBO (from 1.5 to 3 ATA) using different animal models of both global and focal ischemia, where neuronal protection effect was observed even when this therapy was applied up to 12 h after ischemic insult (Li et al. 2005; Lou et al. 2004; Niklas et al. 2004; Wang et al. 2008; Zhang et al. 2005).

There are reports that normobaric hyperoxia administered early in the injury and for a prolonged period of time prevents delayed post-ischemic neuronal death (Kumaria and Tolias 2009). NBO applied during ischemia had been shown to significantly reduce neuronal cell death, and brain infarct volume both in vivo and in vitro inhibits the upregulation of matrix metalloproteinase-2 and attenuate nitric oxide production (Yuan et al. 2010; Kim et al. 2005; Günther et al. 2004). Studies using NBO and HBO after experimental cerebral ischemia provide evidence for blood–brain barrier stabilization, although the effect of NBO was rather weak (Veltkamp et al. 2005). Our results show that NBO applied after ischemia resulted in only a slight reduction in apoptosis and increase in the number of surviving neurons.

The results presented in this paper indicate a more potent protective effect of HBO than NBO and are in agreement with data published by Beynon et al. (2007) showing that even delayed HBO treatment is more effective than early prolonged NBO. Surprisingly, in our experiments, HBA therapy appeared to be more effective than NBO. HBA applied up to 6 h after ischemia resulted in decrease in apoptotic neurons and significant increase in the number of surviving neurons in the CA1 region of the gerbil hippocampus. Only few studies describe the effect of HBA on postischemic survival, and published data show only a slight, if any, protective effect of HBA (Peng et al. 2008; Günther et al. 2004). This makes the results presented in this paper more interesting, since the beneficial effect of HBA was not only limited to a reduced number of dying neurons but also manifested itself in the animals’ behavior. These results may also resolve doubts concerning the effectiveness of oxygen administration by aviators mask in air pressurized chambers (Harch and Neubauer 2009).

Although the neuronal death after ischemic episode is delayed, during the initial postischemic period preceding the death of CA1 neurons, gerbils demonstrate a behavioral deficit, which seems to be linked to hippocampal dysfunction (Duszczyk et al. 2006a). The control gerbils exhibit stereotypical nest-building behavior, characterized by a species-specific shredding of the nest material, in this case a soft paper towel. It is well documented that global brain ischemia in gerbils results in disturbances in nest-building behavior, and this correlates directly with the extent of ischemic morphological damage (Antonawich et al. 1997; Baldwin et al. 1993; Duszczyk et al. 2006a). A typical male gerbil builds a nest within 12–24 h. In our studies, sham-operated animals serving as controls started nest building immediately after placing them in the experimental cage. Animals submitted to 3-min ischemia showed a 2-day delay in the initiation of nest building. It has been suggested that disruption of nest building is not a secondary effect of increased motor activity, which usually accompanies transient global ischemia, but results from delays in habituation and spatial mapping resulting from hippocampal damage (Antonawich et al. 1997; Babcock et al. 1993). Administration of HBO treatment after ischemia significantly improved nest building, which may reflect a protective effect of hyperoxygenation on neurons. Animals which received NBO and HBA treatment after ischemia also showed an improvement in nest-building behavior, which was not significantly different from that observed after HBO. Generally, delivery of oxygen under increased partial pressure within 6 h after ischemia significantly improved animal’s nest-building behavior. The beneficial effect of HBO on neurological deficits graded on the Garcia scale (Garcia et al. 1995) was reported earlier in the rat model of focal cerebral ischemia (Miljkovic-Lolic et al. 2003; Beynon et al. 2007); however, no beneficial effect of NBO was observed (Beynon et al. 2007) and, to our knowledge, there are no reports regarding the effect of HBA on animal behavior after ischemia. Thus, results presented in this paper for the first time show that HBA may not only protect CA1 neurons from ischemia-induced damage but also improve nest-building behavior in gerbils. These results indicate that the increased survival of neurons observed in our experiments correlates with an improved neurological outcome.

There are many mechanisms proposed to explain the beneficial effects of HBO treatments, including increased oxygenation of ischemic and penumbra area in the brain, reduction in blood–brain barrier damage, elevation of autophagic activity, attenuation of nitric oxide production and inhibition of apoptotic protein expression (Ostrowski et al. 2005; Yuan et al. 2010; Veltkamp et al. 2005; Yan et al. 2011). There are reports that supplemental oxygenation applied during reperfusion may result in intensification of injury (Rink et al. 2010). However, increased oxygenation evoked by HBO reduces neuronal death and improves neurological outcome, which was shown not only in our experiments but also after canine cardiac arrest (Rosenthal et al. 2003), circulatory occlusion in cats (Kapp et al. 1982) and cardiopulmonary arrest in pigs (Van Meter et al. 2008). It was recently shown that the improvement in neurological function and reduced neuronal cell death observed after cardiac arrest and reperfusion does not result from increased cerebral oxygen delivery or oxygen consumption (Rosenthal et al. 2003). It is possible that hyperbaric oxygen or hyperbaric treatment itself may trigger more protective mechanisms and the maintenance of brain temperature might be one of them.

Previous studies show that hyperthermia is frequently observed in the first 72 h after resuscitation from cardiac arrest and were associated with poor outcome (Takasu et al. 2001). Similarly, ischemic stroke is usually followed by hyperthermia, resulting from both a stroke-induced inflammatory reaction and disturbances in cell metabolism (Zaremba 2004). Mild therapeutic hypothermia is currently the only therapy that improved survival and brain function after initial resuscitation from cardiac arrest (Janata and Holzer 2009; Sugerman and Abella 2009). It protects the brain after ischemia by decreasing metabolism, inhibiting excitatory amino acid release, and also by attenuation of reactive oxygen species formation and the immune response during reperfusion (Janata and Holzer 2009).

Mongolian gerbils are particularly susceptible to bilateral carotid occlusion, which results in global forebrain ischemia, due to the incomplete circle of Willis (Du et al. 2011). In this model of ischemia, brain temperature often significantly decreases during occlusion but then quickly returns to normal after the onset of recirculation (Zhang et al. 1997; Colbourne et al. 1993). However, at 10–20 min after the start of recirculation, brain temperature increases by 0.7–1 °C and this postischemic hyperthermia is observed for 45–90 min.; thereafter, temperature returns to normal. Brain temperature measurements presented in this paper are mostly in agreement with previous observations. However, in our experiments, the increase in brain temperature lasted for almost 6 h after reperfusion and remained slightly increased to the end of measurements. Previously, we also observed a prolonged (3–4 h) period of post-ischemic hyperthermia in gerbils submitted to 3-min global ischemia (Duszczyk et al. 2005, 2006a). The basis of postischemic temperature increase in the gerbil remains uncertain; however, the hyperthermia described in certain focal ischemia models in rats has been suggested to be caused by altered blood flow in the hypothalamus (He et al. 1999; Zhao et al. 1994), although damage of the blood–brain barrier and development of inflammatory processes may also be an explanation (Veltkamp et al. 2005).

Wood and Gonzales (1996) showed that hyperthermia increases the imbalance between energy supply and demand following ischemia. Temperature-dependent changes in functional neurological outcome, histopathology, intraneuronal calcium accumulation and the levels of enzymes mediating calcium effects, including neuronal excitability, synaptic modulation and release of excitatory neurotransmitters, were demonstrated in number of animal models of cardiac arrest and stroke (Dietrich et al. 1996; Ginsberg and Busto 1998; Wass et al. 1995; Coimbra et al. 1996). These changes may cause further development of postischemic injury of neurons, leading to irreversible lesions. Hyperthermia may also induce additional dysfunction of the blood–brain barrier, facilitating the regional influx of leukocytes as a result of the ischemia-evoked inflammatory reaction (Zaremba 2004). Thus, the prevention of hyperthermia within the first hours after resuscitation from cardiac arrest or stroke is important for preventing further damage to the brain.

The results presented in this paper show that both HBO and HBA treatment applied up to 3 h after ischemia prevents ischemia-evoked increase in brain temperature, especially that observed up to 10 h after reperfusion. Within 72 h, brain temperature of animals treated with hyperbaric therapy remained slightly lower or the same level as before ischemia. Hyperbaric treatment applied 6 h after ischemic insult also prevented the increase in brain temperature, although HBA was less effective than HBO. NBO treatment effectively prevented brain temperature increase only when applied 1 h after ischemia. The results presented in this paper show the correlation between morphological protection, behavioral improvement and attenuation of postischemic hyperthermia. This suggests the causal connection between presented data and indicates a possible key role of brain temperature decrease in beneficial effects of hyperbaric treatments.

It is known from the literature that increased partial pressure of oxygen causes hypothermia. It has been shown in rats that HBO is associated with a significant decrease in body temperature and that this effect is evoked mostly by an increase in the partial pressure of oxygen, and only in small part by heat loss due to pressure alone (Fenton et al. 1993). In our experiments, a decrease in brain temperature during both HBO and HBA was also observed, while only a slight temperature drop was observed in gerbils subjected to NBO. This observation is difficult to explain because the oxygen partial pressure during NBO is two times higher than during HBA (data not shown); however, perhaps the effect of pressurization itself should not be totally excluded. In fact, Fenton et al. (1993) observed a decrease in body temperature in pressure control experiments, where the partial pressure of oxygen at 4 ATA was the same as the partial pressure in air at 1 ATA. Recently, Tsai et al. (2005) found beneficial effects of 8 % oxygen pressurized to 253 kPa (2.5 ATA) in resuscitating rats with experimental heatstroke. The mechanism of this hyperbaric treatment induced hypothermia is still not clear, although the initially proposed involvement of 5-HT_1A_ receptors was excluded (Fenton et al. 1993). These results together with results presented in this paper suggest that the pressurization itself (occurring during HBO and HBA) might be the causal reason for the observed beneficial effects of HBO and HBA.

Concerning the potential mechanisms of pressure-related hypothermia, the suggestion that decrease in CBF caused by high pressure may induce hypothermia and also does not seem to be good explanation because pressure used in presented in this paper experiments (2.5 ATA) only slightly changes CBF (Demechenko et al. 2005). Further, one of the explanations of observed hypothermia may be the increased oxygen supply delivered to the ischemic and penumbra regions of the brain and in consequence improvement in aerobic metabolism of injured regions, at the time window which may guarantee at least partial prevention of neuronal damage (Rockswold et al. 2001). However, Rosenthal et al. (2003) using the animal cardiac arrest model showed no increase in cerebral oxygen delivery and consumption during hyperbaric treatment. It was suggested that probably there is no ongoing ischemia during postischemic hypoperfusion and that energy metabolism may not be limited by oxygen delivery but rather by the activity of aerobic metabolic enzymes (McKinley et al. 1996; Rosenthal et al. 2003).

Hyperbaric reduction in blood–brain barrier damage preventing inflammatory processes and inhibition of neutrophil adhesion to the endothelium are also factors which may contribute in neuronal protection (Buras and Reenstra 2007; Veltkamp et al. 2005). There is now agreement that the best time window for hyperbaric therapy is within the first 6 h after ischemic insult. Our results show that this time window is effective both for HBO and HBA. The best time for NBO application is up to 3 h after ischemia, and during this time, NBO may serve as a useful adjunct therapy widening the time window for reperfusion by as much as 2 h (Kim et al. 2005). However, clinical practice shows that oxygen applied less than 1 h after resuscitation from cardiac arrest results in a dose–dependent association between supranormal oxygen tension and risk of in-hospital death (Kilgannon et al. 2011).

In conclusion, the results presented here show for the first time that not only HBO but also HBA applied between 1 and 6 h after transient forebrain ischemia may prevent ischemia-induced neuronal damage in the CA1 region of the gerbil brain and that this protection is protection is mostly likely due to a pressure-related inhibition of brain temperature increase which typically originates from ischemia. Although early applied NBO also resulted in neuronal protection, this treatment was less effective than delayed HBO or HBA therapy.
